# Identification and Validation of Reference Genes for RT-qPCR Normalization in* Mythimna separata* (Lepidoptera: Noctuidae)

**DOI:** 10.1155/2018/1828253

**Published:** 2018-07-31

**Authors:** Ke Li, Na Xu, Yu Jing Yang, Jin Hui Zhang, Huan Yin

**Affiliations:** College of Life Sciences, Shanxi Normal University, Linfen 041004, China

## Abstract

*Mythimna separata* is a major agricultural pest with seasonal migrating trait in China. Formation and regulation mechanism of migration behavior has resulted in a large number of fundamental researches involving quantitative studies of gene expression in this species. Using appropriate reference gene is critical in RT-qPCR data normalization. A comprehensive study on the reference genes in* M. separata* is lacking. In this paper, expression stabilities of ten candidate reference genes were evaluated in* M. separata* under various biotic and abiotic conditions by employing four different software geNorm, NormFinder, BestKeeper, and the comparative ΔCT method. The comprehensive stabilities ranking of these genes were suggested by RefFinder.* PKG* as a target gene was employed to justify the number of reference genes in four larval tissues and two photoperiod treatments. Results demonstrate that the first three most stable genes were as follows:* EF*,* CypA*, and* β-TUB* for developmental stages;* EF*,* CypA*, and* RPL12* for larval tissues;* EF*,* TBP*, and* β-TUB *for adult tissues*. RPL12*,* β-TUB*, and* EF* for densities;* EF*,* RPL12*, and* GAPDH* for photoperiod treatments;* β-TUB*,* EF*, and* ATPase* for temperature treatments. Stable reference gene combinations may reduce bias in normalization. This work provides for the first time a comprehensive list of appropriate reference genes and facilitates future studies on gene function of* M. separata.*

## 1. Introduction

The oriental armyworm,* Mythimna separata* (Lepidoptera: Noctuidae), is an important agricultural pest in Asia and Australia [1]. In China, it is widely distributed except in the far northwest (Xinjiang). The larvae of the armyworm can inflict devastating damage to more than 104 species of plants in 16 families including food crops (wheat, rice, foxtail millet, and maize) [2]. Each year, the nocturnal moths engage in a seasonal long-distance migration between southern and northern China [3].* M. separata* has been extensively studied as a model migratory species in China [3–5]. The research contents mainly involved environmental, physiological, hormonal, genetic, and molecular factors [3]. The fifth and sixth instars of the larvae (the gluttony period) are the very important stages in which larvae consume vast food to accumulate sufficient nutrition and energy to meet remaining development and migration requirements. Environmental factors such as temperature [6, 7], photoperiod [8], and larval density [9–11] can trigger or inhibit the onset of migration. Moths response to larval environmental conditions [12] and are most likely to take off on a migratory flight the first or second night after adult eclosion [13]. Therefore, molecular studies towards environmental factors and specific developmental stages are the focus of migration research in* M. separata*. Exploring target gene expression profiles in tissues from various environmental conditions or developmental periods will promote a greater understanding of the migration regulation mechanism. Reports on molecular regulation mechanisms of migration for* M. separata *were relatively scarce.

The real-time quantitative polymerase chain reaction (RT-qPCR), as a reliable and rapid method, has been widely applied in gene profiling or expression analysis in organisms. Compared with classic quantitative PCR, this approach offers distinct advantages including high sensitivity, specificity, accuracy, reproducibility, and requirement for less post-PCR processing [14, 15]. However, some variables may influence the accuracy of RT-qPCR, such as total RNA quality, the efficiency of reverse transcription, and primer transcription efficiency [16–19].

Therefore, it is essential to minimize the effect of these variations by RT-qPCR normalization using reference genes [20, 21]. An ideal reference gene should be expressed at a constant level in samples from various cells, tissues, developmental stages, or treatment conditions and does not coregulate with the target gene [21]. To date, the reference genes of several insects have been identified and validated for RT-qPCR normalization among different biotic or abiotic conditions, e.g.,* Apis mellifera *(Hymenoptera: Apidae) [22],* Bombyx mori* (Lepidoptera: Bombycidae) [23],* Schistocerca gregaria* (Orthoptera: Acrididae) [24],* Bactrocera dorsalis* (Diptera: Tephritidae) [25],* Tribolium castaneum* (Coleoptera: Tenebrionidae) [26],* Spodoptera litura* (Lepidoptera: Noctuidae) [27],* Spodoptera exigua* (Lepidoptera: Noctuidae) [28],* Helicoverpa armigera* (Lepidoptera: Noctuidae) [29],* Anastrepha obliqua* (Diptera: Tephritidae) [30], and* Chilo suppressalis *(Lepidoptera: Crambidae) [31]. These findings demonstrate that there is no single gene that meets all requirements of each experimental condition. Therefore, validation of specific reference gene needs to be conducted for each species and experimental background before using them. Up to now, the researches on identification and evaluation of suitable reference genes for RT-qPCR gene expression normalization in* M. separata *are scare.

In the current paper, 10 commonly used reference genes were examined in* M. separata* to normalize RT-qPCR data. The expression profile of protein kinase G gene (*PKG*) was applied to further validate the selected reference genes. The aims are to identify and evaluate the expression stability of the reference genes under different biotic (development stage, tissue, and density) and abiotic (photoperiod and temperature) conditions. This will facilitate future researches on gene regulation involved in* M. separata* migration.

## 2. Materials and Methods

### 2.1. Insects

The insects were initially obtained from a culture at the Biorational Pesticides Research and Development Center, Northwest A&F University, China, and maintained under a L14: D10 regime at 23 ± 1°C and 70 ± 10% relative humidity (RH). In each treatment, the colonies were derived from one batch of eggs laid by a single male/female pair. The larvae were immediately reared in one 850-ml jar from the day of hatching with fresh maize seedling. The samples were dissected under light CO2 aesthesia and immediately immersed in liquid nitrogen and stored at −80°C until used.

### 2.2. Biotic Factors

#### 2.2.1. Developmental Stages

Samples of brain were collected from 8 developmental stages, including 3 larval stages (from fourth instar to sixth instar; L4–L6), 1 prepupal stage (PP), 3 pupal stages (P1; P5; P9), and 1 adult (A0). In the larval stage, two-day-old larvae from each instar stage were selected in this paper. The newly formed pupa is designated as day P0. Subsequent days of pupal development are counted as P1–P11. The freshly formed adult is termed A0. The samples were in triplicate, 20 larvae in each repetition, and stored as just described.

#### 2.2.2. Larval Tissues

Four larvae tissues (brains, epidermises, fat bodies, and alimentary canals) were obtained from second day larvae of 5th instar stage. Each sample was repeated three times, 15 larvae in each repetition. All samples were stored as mentioned earlier.

#### 2.2.3. Adult Tissues

Six adult tissues, including male brains, female brains, testes, ovaries, male fat bodies, and female fat bodies, were dissected from one-day-old adults. For each tissue, 15 insects were collected. Each sample was repeated three times. All samples were stored as mentioned earlier.

#### 2.2.4. Densities

The larvae were immediately separated after hatching and formed four larval densities regimes including 1, 10, 20, and 30 larvae/850 ml jar and then were reared with fresh maize seedlings under a L14:D10 cycle at 23 ± 1°C and 70 ± 10% relative humidity (RH). Each density regime had three repetitions, 20 larvae in each repetition. Brains were obtained from the fifth instar larvae and stored as described above.

### 2.3. Abiotic Factors

#### 2.3.1. Photoperiod Treatments

Colonies newly hatched were reared at a density of 10 larvae per 850-ml jar and subjected to two different photoperiod regimes (8L: 16D and 16L: 8D) at 23 ± 1°C and 70 ± 10% relative humidity (RH). Each photoperiod treatment had three repetitions, 20 larvae in each repetition. Brains were obtained from 5th instar larvae and stored as mentioned above.

#### 2.3.2. Temperature Treatments

As photoperiod treatment, larvae after hatching were reared at a density of 10 larvae/850 ml jar and were exposed at two temperature regimes (18°C and 25°C) under 70 ± 10% RH and L14: D10 photoperiod. Each temperature treatment had three repetitions, 20 larvae in each repetition. Brains were obtained from 5th instar larvae and stored as mentioned previously.

### 2.4. RNA Extraction and cDNA Synthesis

Total RNA was extracted with RNAiso Plus (Takara Bio Inc., Dalian, China) according to the manufacturer's instructions. First-strand cDNA was synthesized from 1 *μ*g of total RNA using the PrimeScript®RT reagent Kit (Perfect Real-Time) (Takara Bio Inc.) with gDNA Eraser following the manufacturer's protocols. The synthesized cDNA was stored at −80°C.

### 2.5. Reference Gene Selection and Primer Design

Ten commonly used reference genes were selected (Table 1), including 18S ribosomal (*18S*), 28 ribosomal (*28S*), ribosomal protein L12 (*RPL12*), glyceraldehyde-3-phosphate dehydrogenase (*GAPDH*), cyclophilin A (*CypA*), beta-actin (*β- ACT*), beta-tubulin (*β-TUB*), vacuolar V-type H (+) ATPase (*ATPase*), TATA-box-binding protein (*TBP*), and elongation factor 1 alpha (*EF*). The gene-specific primers for RT-qPCR were designed with Primer-BLAST [32] based on the gene sequences obtained from the GenBank of NCBI. All primers were synthesized by Beijing AuGCT Biotechnology Co., Ltd (Beijing, China). Specificity of RT-qPCR amplifications was confirmed for each primer pair by melting curve analysis and 1.2% agarose gel electrophoresis. Amplification efficiency for each pair of primers was determined by the slopes of standard curve generated using 10-fold series dilution of the cDNA and calculated by applying the formula* E *(%) = (10^(−1/slope)^-1) × 100% [33].

### 2.6. Real-Time RT-qPCR and Expression Stability Analysis

RT-qPCR was conducted in an iQ™5 Multicolor Real-time PCR Detection system (Bio-Rad, Hercules, CA, USA). The amplification reactions were performed in a volume of 20 *μ*L containing 8 diluted cDNA, 10 *μ*L SYBR Premix EX Taq polymerase (Takara Bio Inc.), and 1 *μ*L of each 10 *μ*M primer. The cycling conditions were 95°C for 30s, 40 cycles of 95°C for 30 s, and 60°C for 30 s, followed by a dissociation-curve program from 60°C to 95°C for verification of PCR amplicons specificity. Standard curves were created with 10-fold dilution series of pooled cDNA for each treatment using the linear regression model. For both biotic and abiotic conditions, all reactions were performed in three technical replicates. All biological replicates were used to calculate the average CT value.

The expression stabilities of the candidate reference genes were evaluated by geNorm [34], NormFinder [35], BestKeeper [36], and the comparative ΔCT method [37]. RefFinder, a web-based analysis tool (http://150.216.56.64/referencegene.php), was finally applied to evaluate and screen the optimal reference genes by integrating the results obtained from the above four programs. In addition to the stability evaluation, the optimal number of reference genes is determined with the geNorm software by calculating a normalization factor (NF). geNorm calculates the pairwise variation V_n_/V_n+1_ between two sequential normalization factors NF_n_ and NF_n+1_ containing and increasing number of reference genes. If V_n_/V_n+1_ < 0.15 the inclusion of an additional reference gene is not required and the recommended number of reference genes is given by n [34]. Given the case that all pairwise variation values are above the proposed 0.15 cut-off value, the optimal number of reference genes for normalization, the lowest pairwise variation is recommended [34, 38].

cGMP-dependent protein kinase (*PKG*) gene was used to evaluate the validity of selected reference genes under biotic (larval tissues) and abiotic (photoperiod) conditions. All the experiments were performed in triplicate. Its expression levels were determined according to the CT value based on the 2^−ΔΔCT^ method [39]. The relative expression levels in the four larval tissues (brains, epidermises, alimentary canals, and fat bodies) were analyzed using a one-way analysis of variance (ANOVA) followed by Tukey's* post hoc* test. Significance values were set to* P *< 0.05.

## 3. Results

### 3.1. Primer Specificity and PCR Efficiency

Ten candidate reference genes (Table 1) were selected to normalize the gene expression levels in* M. separata. *The qPCR primer pairs for these reference genes were specific according to the analysis of a single sharp peak in melting curve and the presence of specific bands of the expected size in the agarose gel electrophoresis. Each primer pairs amplification efficiency ranged from 96.8% to 108.9% and correlation coefficients (R2) were not less than 0.99 (Table 1), which were within an acceptable range for qPCR.

**Figure 1:**
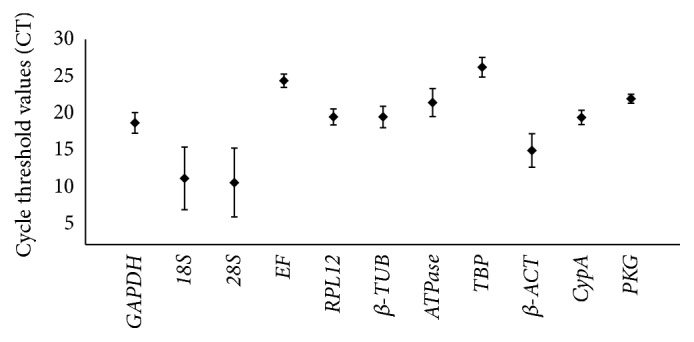
Expression profiles of candidate reference genes and target genes in different samples of* M. separata.* Expression levels are documented as cycle threshold (CT) values of candidate reference genes used in this study. The black dots indicate the mean value of replicated samples, while the bars indicate the standard deviation of the mean.

A broad range of CT values from 10.71 (*28S*) to 25.91 (*TBP*) in RT-qPCR exhibited all reference genes expression levels across all treatments (Figure 1).* 18S* (mean CT value) and* 28S* (mean CT value) were expressed at the highest levels and* EF* (mean CT value) and* TBP* (mean CT value) at the lowest levels. The six remaining reference genes were expressed at moderate levels (mean CT values of 18.87, 19.84, 19.83, 21.57, 15.82, and 18.7 for* GAPDH*,* RPL12*,* β-TUB*,* ATPase*,* β-ACT*, and* CypA,* respectively).

### 3.2. Stability of Candidate Reference Genes under Biotic Conditions

#### 3.2.1. Developmental Stages

**Figure 2:**
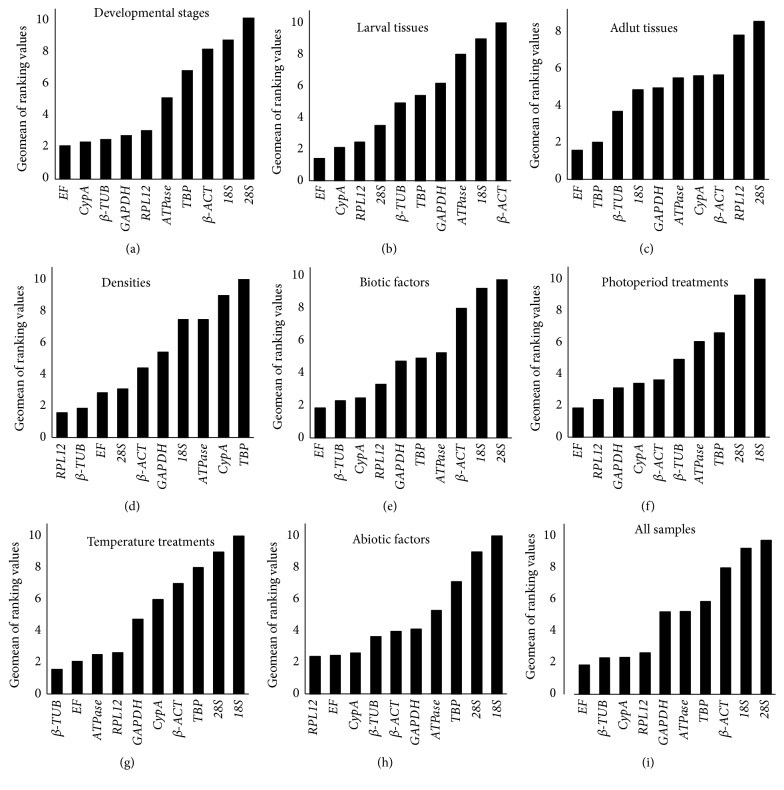
Expression stabilities of candidate reference genes in different samples. The average expression stabilities of the reference genes as calculated by the Geomean method of RefFinder. A lower Geomean of ranking value denotes more stable expression. (a) Different developmental stages; (b) larval tissues; (c) adult tissues; (d) densities; (e) biotic factors; (f) photoperiod treatments; (h) temperature treatments; (g) abiotic factors; (i) all samples.

**Figure 3:**
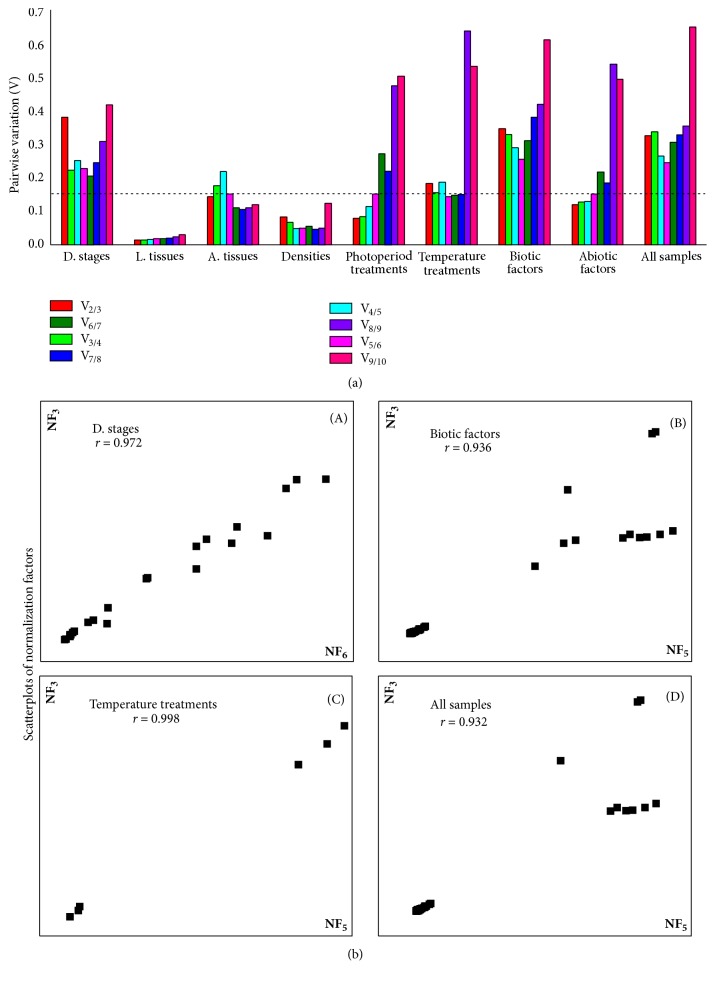
Optimal number of reference genes for normalization in* M. separata*. (a) Pairwise variation (V_n_ / V_n+1_) was analyzed between the normalization factors NF_n_ and NF_n+1_ to determine the optimal number of reference genes required for accurate normalization. A value < 0.15 denotes that additional reference genes will not markedly improve normalization. (b) Correlation between the NF of most three stable and optimal number endogenous control. Pearson's correlations between the NFs of three endogenous control genes (NF_3_) and optimal number (y-axis) of endogenous control genes (NF_opt_) for (A) developmental stages, (B) temperature treatments, (C) biotic factors, and (D) all samples.

The stability ranking order of the first 3 most stable genes obtained from four programs were inconsistent (Table 2). The least stable genes determined by four programs were* 18S*,* 28S*, and* β-ACT*. According to RefFinder, the stability ranking of the reference genes from the most stable to the least stable across different developmental stages is as follows:* EF *>* CypA *>* β-TUB *>* GAPDH *>* RPL12 *>* ATPase *>* TBP* >* β-ACT *>* 18S *> 28S (Figure 2(a)). The geNorm analysis showed that all pairwise variation values were above the proposed 0.15 cut-off value (Figure 3(a)). The lowest pairwise variation was shown at V_6/7_ (the variation between the normalization factors of six genes in relation to seven genes); thus, six genes are recommended as reference genes for normalization. It is relatively impractical to use excessive numbers of endogenous control genes for normalization. The use of the three most stable control genes was considered to be adequate for normalization of RT-qPCR [34, 38]. To verify this recommendation, the correlation of NF values between the three most stable genes and the optimal number of genes was calculated. As shown in Figure 3(b)-(A), there is a good correlation between two NF measures (proposed number, three; the theoretical optimal number, six) for developmental stages (*r* = 0.972). This result supports that the three most stable internal control genes are sufficient for an accurate normalization of developmental stages data. Thus, the normalization with the combination* EF*,* CypA*, and* RPL12* should be suggested by geNorm for a suitable normalization in the different developmental stages. And geNorm is basically in line with RefFinder identifying three out of ten most stable genes (Figure 2(a)).

#### 3.2.2. Larval Tissues

*18S* and* β-ACT *were identified as the least stable genes by the four programs in different tissues (Table 2(a)). The four programs revealed that* EF* was the most stable gene (Table 2(a)). According to RefFinder, the stability ranking of reference genes from the most stable to the least stable gene under various tissues was as follows:* EF *>* CypA* >* RPL12 *> 28S >* β-TUB *>* TBP *>* GAPDH *>* ATPase* >* 18S *>* β-ACT* (Figure 2(b)). In contrast with developmental stages, all pairwise variation values obtained by the geNorm were below the proposed 0.15 cut-off value (Figure 3(a)). The normalization with the combination* EF* and* RPL12* was proposed by geNorm in the various tissues samples. It is roughly identical to the analysis result of RefFinder software calculating three most stable genes.

#### 3.2.3. Adult Tissues

*β-ACT *was identified as the least stable genes by the three programs in different tissues (Table 2(a)) except BestKeeper. The four programs, except BestKeeper, revealed that* EF* and* TBP *were the most stable genes (Table 2(a)). According to RefFinder, the stability ranking of reference genes from the most stable to the least stable gene under various tissues was as follows:* EF *>* TBP *>* β-TUB *>* 18S *>* GAPDH *>* ATPase* >* CypA* >* β-ACT *>* RPL12 *> 28S (Figure 2(c)). V_2/3_ pairwise variation value obtained by the geNorm was below the proposed 0.15 cut-off value (Figure 3(a)). Therefore, the normalization with the combination* EF* and* TBP* was proposed by geNorm in the various tissues samples. It is consistent with the calculational result of RefFinder software proposing three out of ten most stable reference genes.

#### 3.2.4. Densities

It is similar to the results determined by NormFinder in which the stability ranking identified by geNorm revealed that* RPL12, β-TUB*, and* 28S *were the most stable genes (Table 2(b)).* CypA* and* TBP* were the least stable genes identified by four programs (Table 2(b)). According to RefFinder, the stability ranking of the reference genes from the most stable to the least stable gene across density was as follows:* RPL12 *>* β-TUB *>* EF *> 28S >* β-ACT *>* GAPDH *>* 18S *>* ATPase *>* CypA* >* TBP *(Figure 2(d)). The geNorm analysis showed that all pairwise variation values were below the proposed 0.15 cut-off value (Figure 3(a)). The combination of two genes,* RPL12* and* β-TUB*, is recommended by geNorm for a suitable normalization in the density samples. It is similar to the analysis result by RefFinder software recommending the two most stable control genes.

#### 3.2.5. All Biotic Samples

The stability ranking of the reference genes proposed by NormFinder was closely similar to the result revealed by the ΔCT method (Table 2(b)), which identified* β-TUB* and* EF* as the most stable genes. Three genes (*β-ACT*,* 18S*, and* 28S*) were the least stable genes revealed by four programs (Table 2(b)). The geNorm showed* RPL12* and* CypA* were the most stable genes, whereas,* EF* and* TBP* determined by BestKeeper were the most stable genes (Table 2(b)). According to RefFinder, the stability ranking of the reference genes from the most stable to the least stable gene under all biotic samples was as follows:* EF *>* β-TUB *>* CypA* >* RPL12 *>* GAPDH *>* TBP *>* ATPase* >* β-ACT *> 18S >* 28S *(Figure 2(e)). All pairwise variation values calculated by the geNorm were above the proposed 0.15 cut-off value (Figure 3(a)). According to the lowest pairwise variation appearing at V_5/6_, five genes are proposed as reference genes for normalization. As for the developmental stages, the correlation of NF values between NF_3_ and NF_5_ was analyzed. As shown in Figure 3(b)-(B), there is a good correlation between two NF measures for all biotic samples (*r* = 0.936). This result supports that the three most stable reference genes may meet an accurate normalization for all biotic samples. Thus, the normalization with* RPL12*,* CypA*, and* EF *was proposed by geNorm across all biotic samples. This result is in general accordance with that of RefFinder.

### 3.3. Stability of Candidate Reference Genes under Abiotic Conditions

#### 3.3.1. Photoperiod Treatments

Gene stability ranking order determined by BestKeeper was closely similar to the results revealed from the ΔCT method, which revealed* EF*,* RPL12*, and* CypA* to be the most stable genes (Table 3). The most stable genes identified by geNorm were* GAPDH*,* β-TUB*, and* CypA*, whereas those identified by NormFinder were* EF*,* β-ACT*, and* ATPase *(Table 3). The four programs revealed* 18S* and* 28S* to be the least stable genes (Table 3). According to RefFinder, the stability ranking of the reference genes from the most stable to the least stable gene across photoperiod was as follows:* EF *>* RPL12 *>* GAPDH *>* CypA *>* β-ACT *>* β-TUB *>* ATPase *>* TBP*> 28S >* 18S *(Figure 2(f)). For this treatment, the first V-value < 0.15 showed at V_2/3_ (Figure 3(a)), suggesting that two reference genes were sufficient for reliable normalization. Thus, the normalization with the combination of* GAPDH* and* β-ACT *was proposed by geNorm in all photoperiod treatments.

#### 3.3.2. Temperature

Gene stability ranking results of most stable genes determined by geNorm was very similar to the results obtained from NormFinder, which showed* β-TUB*,* ATPase*, and* EF* to be the most stable genes (Table 3). BestKeeper and the ΔCT method were closely similar in the ranking results, which exhibited that* RPL12*,* EF*, and* β-TUB *were the most stable genes (Table 3).* 28S* and* 18S* determined by the four programs were the least stable genes. According to RefFinder, the gene stability ranking from the most stable to the least stable across temperature was as follows:* β-TUB *>*EF *>* ATPase *>* RPL12 *>* GAPDH *>* CypA *>* β-ACT *>* TBP *> 28S >* 18S* (Figure 2(g)). The geNorm analysis showed that the pairwise variation values of V_5/6_, V_6/7_, and V_7/8_ were the cut-off value of 0.15 (Figure 3(a)). According to the lowest pairwise variation shown at V_5/6_, five genes are proposed as reference genes for normalization. As previously described, the correlation of NF values between NF_3_ and NF_5_ was calculated. As shown in Figure 3(b)-(C), there is a good correlation between two NF measures for all biotic samples (*r* = 0.998). It supports that the three most stable reference genes may suit an accurate normalization for temperature treatments. Thus, the normalization with* β-TUB*,* EF*, and* ATPase* was recommended by geNorm across temperature treatments. This result is consistent with that of RefFinder.

#### 3.3.3. All Abiotic Samples

The results of gene stability ranking obtained from BestKeeper and the ΔCT method were similar, which revealed that* EF*,* RPL12*, and* β-TUB *were the most stable genes (Table 3). Four programs identified* 28S* and* 18S* as the least stable genes.* β-ACT and CypA* determined by geNorm were the most stable genes; however,* β-TUB and ATPase* recommended by NormFinder were the most stable genes (Table 3). According to RefFinder, the gene stability ranking from the most stable to the least stable across temperature was as follows:* RPL12 *>* EF *>* CypA *>* β-TUB *>* β-ACT* >* GAPDH *>* ATPase *>* TBP *> 28S >* 18S* (Figure 2(h)). Pairwise variation values of V_2/3_, V_3/4_, and V_4/5_ calculated by the geNorm were below the proposed 0.15 cut-off value (Figure 3(a)). Thus, the combination of two reference genes,* CypA* and* β-ACT*, proposed by geNorm was suitable for normalizing RT-qPCR data in all abiotic samples.

### 3.4. All Samples

The stability ranking results determined by NormFinder and the ΔCT method were similar. These programs revealed that* β-TUB*,* EF*, and* CypA *were the most stable genes (Table 4).* 18S* and* 28S* were identified by the four programs as the least stable genes (Table 4). The stability ranking of the first four genes determined by BestKeeper was inconsistent with that of the ΔCT method (Table 4). According to RefFinder, the gene stability ranked from the most stable to the least stable across all samples was as follows:* EF *>* β-TUB* >* CypA *>* RPL12 *>* GAPDH *>* ATPase *>* TBP *>* β-ACT *>* 18S* >* 28S* (Figure 2(i)). All pairwise variation values exhibited by geNorm were above the proposed 0.15 cut-off value (Figure 3(a)). The value at V_5/6_ was lowest in all pairwise variations; five are proposed as reference genes for normalization. As described above, the correlation of NF values between NF_3_ and NF_5_ was calculated, and there is a good correlation between two NF measures for all biotic samples (*r* = 0.998) (Figure 3(b)-(D)). So, the three most stable reference genes (*CypA*,* RPL12*, and* EF*) may meet an accurate normalization for all sample. This result is basically consistent with that of RefFinder.

## 4. Validation of Reference Gene Selection

**Figure 4:**
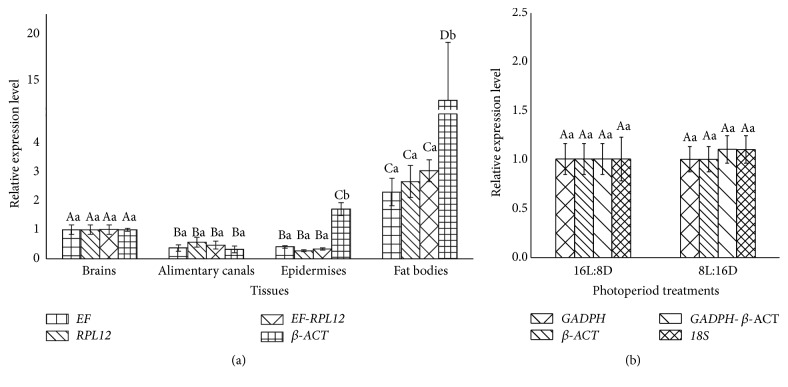
Validation of the gene stability measures. Expression levels of a target gene,* PKG*, in four larval tissues (a) and two photoperiod treatments (b) were tested using different normalization factors. Bars represent the means and standard deviations of three biological replicates. Means followed by the same uppercase letters indicate nonsignificant differences among tissues or photoperiod treatments within each* PKG* expression normalized by reference gene or combination, and the same lowercase letters show nonsignificant differences among* PKG* expression normalized by reference gene or combination within each tissues or photoperiod treatments (*P* > 0.05).

To assess the validity of selected reference genes, the expression profiles of* PKG* under four larval tissues and two photoperiod treatments as a sample were normalized using single reference gene or gene combinations recommended by geNorm (Figure 2) as follows: two most stable reference genes, respectively, the combination of two most stable reference genes, and the least stable gene. Among four various larval tissues, the results of normalizing* PKG* expression were similar when, respectively, using* EF or RPL12* (the most stable reference gene) and the combination* EF and RPL12* (the most stable two) (Figure 4(a)). However, the difference of* PKG* expression among four tissues was significantly different using* β-ACT* normalizing PKG expression and the expression levels of* PKG* normalized using* β-ACT* (least stable) were 7.7-fold to 41.1-fold higher than those of* PKG* normalized using the other two reference genes or gene combinations in fat bodies and 3-fold to 9.2-fold in epidermises.* PKG* expression normalized by* GAPDH or β-ACT* (the most stable reference gene), the combination of* GAPDH and β-ACT *(the most stable most), and 18S (the least stable) (Figure 4(b)) was higher in 8L:16D than that in 16L:8D, but the difference was not statistically significant (*P*>0.05).

## 5. Discussion

RT-qPCR is a routine technique widely used to quantify mRNA levels of target genes. Its accuracy and reliability rest with the reference gene used as endogenous control. However, to date, there are no universal reference genes that are stably expressed in all types of samples under various treatment conditions. Therefore, the evaluation of reference genes must be conducted prior to gene expression profiling experiments. In this study, we firstly examined the validation of ten reference genes commonly applied in all kinds of organisms in* M. separata* with four algorithms (geNorm, NormFinder, BestKeeper, and the ΔCT method) under various biotic and abiotic conditions. The ranking orders of investigated reference genes across the four programs were varied based on various conditions (Tables 2(a), 2(b), 3, and 4). RefFinder, a web-based algorithm, integrates all data obtained from the four programs mentioned above and determines the stability of selected reference genes by calculation of Geometric Mean values, and those with the lower GM values are identified as more stable genes. RefFinder proposed that* EF*,* β-TUB*,* CypA*, and* RPL12* are the most stable reference genes for* M. separata* under all samples (Figure 2(i)).

*Elongation factor-1 alpha* (*EF*), a GTP-binding protein that catalyzes the binding of aminoacyl-transfer RNAs to the ribosome [40], was the most stable reference gene for all biotic factors (developmental stages, tissues, and densities) proposed by RefFinder. The stability of* EF* in two biotic factors (developmental stages and tissues) is in accordance with reference gene analyses in* Drosophila melanogaster* [41], Orthoptera [42], Hymenoptera [43], and* Plutella xylostella* [44] and is counter to that in* S*.* exigua* [28]. Under temperature treatments, the ranking of* EF* in* M. separata *is inconsistent with that in* P. xylostella* [44] and* H. armigera* [29], in which* EF *was one of the least stable genes.* EF* was relatively stable across different treatments in* M. separata*; however, its expression level was low, with a mean CT of 24.49 (Figure 1). Therefore, the expression level of target gene will decide whether EF may be used as a control gene.

*CypA* is a peptidyl-prolyl cis/trans isomerase originally identified as the target of the immunosuppressive drug cyclosporine A [45]. Its applications as reference gene in insects were relatively spares. In* M. separata*, the expression stabilities across developmental stages and tissues were higher than those in other conditions. This result was similar to the stabilities of* CypA* in* Hippodamia convergens* [46]. However, it was the least under the developmental stages and tissues in* Danaus plexippus *(L.) [47]. More remarkably, the expression of* CypA* was unstable among four densities (Figure 2(d)). Previous study has examined that* M. separata *larvae reared at high density show higher resistant to nucleopolyhedrovirus (NPV) than those reared at low density [48].* CypA* was also reported to regulate several severe viruses in human [49]. So, further studies are needed to determine whether* CypA* is involved in the regulation of resistance to NPV in* M. separata *while controlling for larval density.

Ribosomal proteins (RPs) are a large group of proteins which, in conjunction with rRNA, make up the ribosomal subunits involved in the cellular process [50]. In mice (C57B16, 10-12 weeks old), ribosomal protein genes exhibited important tissue-dependent variation in mRNA expression and cannot be considered as true reference genes [51]. In the present study,* RPL12* gene is identified as being the most stable gene under densities and photoperiod treatments and being of relative higher stable ranking in developmental stages, larval tissues, and temperature treatments (Figure 2). Previous researched also exhibited that RP genes were stable under varied treatments in insects, as* RP49* in the different developmental stages of* B. mori* [23],* PRL10*, and* RPL7* in the all tissue sample sets of* S. exigua* [28],* RPL15* under different larvae tissues and* RPL32* under all tested samples in* H. armigera* [29], and* RPL18* in developmental stages in* A*.* obliqua *[30]. Although the number of ribosomal proteins is large, no single gene has been identified to show expression stability across all biotic and abiotic treatments up to now.

*18S* and* 28S*, ribosomal RNAs, were suggested by related studies to be applicable reference genes, due to the fact that their transcript is less affected by treatments that significantly alter mRNA expression [52–54]. In this study, 18S and 28S exhibited the least stable expression at the different developmental stages, photoperiod treatments, and temperature treatments. This result conflicts with that in* H. armigera* [29], but identifies with the findings of* P. xylostella* [44]. These results further prove that it is necessary to validate the expression stability of reference genes.

Further, the expression of* PKG *was conducted in different tissues and in response to photoperiod stress for validating the reference genes. Although the results demonstrated that the expression trends in different tissues were accordant using various reference gene or gene combinations.* PKG *transcript was not induced by photoperiod. However, the results showed that using unstable reference genes may generate the wrong interpretation, and stable reference gene combinations may reduce bias in normalization (Figure 4(a)). Therefore, the validation of candidate reference genes should be conducted to accurately estimate target gene expression, and two or three gene combinations should be used for data normalization.

## 6. Conclusion

In the present study, ten candidate reference genes for normalizing RT-qPCR data in* M. separata* were selected and validated for their expression stability under various biotic and abiotic conditions. Results obtained from RefFinder exhibited that the first three most stable gene were as follows (Table 5):* EF*,* CypA,* and* β-TUB* for developmental stages;* EF*,* CypA*,* and RPL12 *for larval tissues;* EF*,* TBP*, and* β-TUB* for adult tissues;* RPL12*,* β-TUB*, and* EF *for densities;* EF*,* RPL12*, and* GAPDH* for photoperiod treatments;* β-TUB*,* EF*, and* ATPase* for temperature treatments. The results from expression profiles of* PKG* normalized by selected gene(s) suggest that stable reference gene combinations may reduce bias in normalization. This work will facilitate future studies on gene function researches of* M. separata.*

## Figures and Tables

**Table 1:** Primer pairs of candidate reference genes used for RT-qPCR analysis.

Gene symbol	Gene name	Accession number	Sequence	Product length (bp)	Primer efficiency (%)	R^2^
*GAPDH*	Glyceraldehyde-3-phosphate dehydrogenase	HM0055756.1	F: CGCTACAGTCGTTGCCATCA	126	105.8	0.998
			R: ACGACGAGGAAGCCATCTTG			
*18S*	18S ribosomal	MG654665	F: GGCCGTTCTTAGTTGGTGGA	99	106.1	0.998
			R: AGCCACGCACACCTAAATGA			
*28S*	28S ribosomal	MG654666	F: GCAGCGGAACCGTTTCAATA	134	98.80	1.000
			R: ATGGAACTCGAACGCTCAGG			
*EF*	Elongation factor1 alpha	KR869784.1	F: AAGAAATCTGCCCGCGGTAT	72	102.1	0.991
			R: TGCGGTTTAGCGATGGAAGT			
*RPL12*	Ribosomal protein L12	MG665388	F: ACGTTTGATTGCAGTTCGCC	102	100.9	0.995
			R: TTAGGCGGCATTGTGCTGTA			
*β-TUB*	Beta-Tubulin	EU234504.1	F: CGAGCTTGTGGATTCCGTCT	91	108.9	0.992
			R: GCCGAGAGAGTGTGTGAGTT			
*ATPase*	ATP synthase subunit H	KC683729.1	F: AACAACCCTCGATGCTAGGC	98	102.5	0.997
			R: CCGACACCGTCTTGTAGGTC			
*TBP*	TATA box binding protein	MG657270	F: GGCACTACAACAAGCACATGG	86	98.3	0.994
			R: CTCGTGGCCGTACCATTCTG			
*β-ACT*	Beta-Actin	GQ856238.1	F: GTCCTACGAACTTCCCGACG	95	96.8	0.997
			R: CCATACCCAGGAATGAGGGC			
*CypA*	Cyclophilin A	HM113489.1	F: GCTCAGAACAGGTGTGACCC	87	101.1	0.998
			R: GTGGAGCCCTTGTAACCGAA			
*PKG *	cGMP-dependent protein kinase	GQ844298	F: TTACCGGCTCACCACCTTTC	182	99.1	0.998
			R: CTCTGTGATGCCACCTCGTT			

**Table:** (a) Expression stabilities of the candidate reference genes under different biotic conditions

Biotic condition	Reference gene	geNorm		Normfinder		BestKeeper		△Ct	
		Stability	Rank	Stability	Rank	Stability	Rank	Stability	Rank
Developmental stages	*GAPDH*	1.138	5	1.002	4	0.88	1	1.94	4
	*18S*	1.876	9	2.288	8	2.72	9	2.89	9
	*28S*	2.364	10	4.168	10	4.12	10	4.32	10
	*EF*	0.901	3	0.286	1	1.04	3	1.77	2
	*RPL12*	0.375	1	1.186	5	1.06	4	1.96	5
	*β-TUB*	0.964	4	0.335	2	1.12	5	1.73	1
	*ATPase*	1.26	6	0.867	3	1.17	6	2.01	6
	*TBP*	1.377	7	1.868	7	1.2	7	2.38	7
	*β-ACT*	1.57	8	2.321	9	1.59	8	2.71	8
	*CypA*	0.375	1	1.197	6	0.89	2	1.93	3
Larval tissues	*GAPDH*	0.944	6	1.564	7	0.79	5	1.91	7
	*18S*	1.612	9	1.898	9	1.96	9	2.16	5
	*28S*	0.626	3	1.213	5	0.62	2	1.58	9
	*EF*	0.528	1	0.577	2	0.28	1	1.35	2
	*RPL12*	0.528	1	1.043	4	0.62	3	1.52	3
	*β-TUB*	1.144	7	0.642	3	1.12	7	1.53	4
	*ATPase*	1.41	8	1.707	8	1.84	8	2.1	8
	*TBP*	0.674	4	1.505	6	0.84	6	1.77	6
	*β-ACT*	1.761	10	2.128	10	2.01	10	2.35	10
	*CypA*	0.772	5	0.292	1	0.67	4	1.33	1
Adult tissues	*GAPDH*	0.888	7	0.629	3	1.27	7	1.02	4
	*18S*	0.815	6	0.643	5	1.17	3	1.03	6
	*28S*	0.949	8	0.982	8	1.68	9	1.23	9
	*EF*	0.368	1	0.533	1	1.24	6	0.92	1
	*RPL12*	0.663	5	1.002	9	1.76	10	1.2	8
	*β-TUB*	0.386	3	0.629	4	1.18	5	0.97	3
	*ATPase*	1.008	9	0.867	7	0.97	2	1.13	7
	*TBP*	0.368	1	0.534	2	1.18	4	0.93	2
	*β-ACT*	1.085	10	1.244	10	0.83	1	1.39	10
	*CypA*	0.522	4	0.727	6	1.43	8	1.03	5

**Table:** (b) Expression stabilities of the candidate reference genes under different biotic conditions

Biotic condition	Reference gene	geNorm		Normfinder		BestKeeper		△Ct	
		Stability	Rank	Stability	Rank	Stability	Rank	Stability	Rank
Densities	*GAPDH*	0.301	6	0.305	6	0.24	4	0.51	6
	*18S*	0.337	7	0.436	8	0.43	7	0.55	8
	*28S*	0.229	3	0.158	3	0.24	5	0.42	2
	*EF*	0.265	4	0.171	4	0.13	1	0.47	4
	*RPL12*	0.16	1	0.017	1	0.2	2	0.43	3
	*β-TUB*	0.16	1	0.031	2	0.27	6	0.42	1
	*ATPase*	0.36	8	0.342	7	0.44	8	0.51	7
	*TBP*	0.566	10	1.244	10	0.82	10	1.27	10
	*β-ACT*	0.277	5	0.172	5	0.23	3	0.47	5
	*CypA*	0.391	9	0.519	9	0.57	9	0.62	9
Biotic samples	*GAPDH*	1.116	5	1.039	5	1.16	4	1.97	5
	*18S*	1.987	9	2.624	9	3.08	10	3.07	9
	*28S*	2.315	10	3.332	10	2.72	9	3.63	10
	*EF*	0.837	3	0.872	2	0.91	1	1.81	2
	*RPL12*	0.646	1	1.045	6	1.25	5	1.89	4
	*β-TUB*	0.95	4	0.39	1	1.43	7	1.75	1
	*ATPase*	1.411	7	0.996	3	1.31	6	2.03	6
	*TBP*	1.284	6	1.958	7	1.04	2	2.42	7
	*β-ACT*	1.669	8	2.232	8	2.36	8	2.72	8
	*CypA*	0.646	1	1.013	4	1.05	3	1.85	3

**Table 3:** Expression stabilities of the candidate reference genes under different abiotic conditions.

Abiotic condition	Reference gene	geNorm		Normfinder		BestKeeper		△Ct	
		Stability	Rank	Stability	Rank	Stability	Rank	Stability	Rank
Photoperiod	*GAPDH*	0.179	1	1.657	6	0.58	4	1.97	4
	*18S*	2.504	10	5.034	10	5.08	10	5.07	10
	*28S*	1.863	9	3.608	9	3.94	9	3.96	9
	*EF*	0.581	6	0.059	1	0.27	2	1.82	1
	*RPL12*	0.293	4	1.188	4	0.25	1	1.83	2
	*β-TUB*	0.963	7	0.117	2	1.31	7	2.09	6
	*ATPase*	1.169	8	0.117	3	1.43	8	2.14	7
	*TBP*	0.408	5	0.177	8	0.97	6	2.32	8
	*β-ACT*	0.179	1	2.177	7	0.61	5	1.98	5
	*CypA*	0.228	3	1.696	5	0.42	3	1.88	3
Temperature	*GAPDH*	0.704	5	1.396	5	0.77	5	1.96	4
	*18S*	2.689	10	5.316	10	4.99	10	5.36	10
	*28S*	2.021	9	5.064	9	4.79	9	5.17	9
	*EF*	0.43	3	0.173	3	0.21	1	1.86	2
	*RPL12*	0.537	4	0.669	4	0.33	3	1.85	1
	*β-TUB*	0.181	1	0.09	1	0.32	2	1.94	3
	*ATPase*	0.181	1	0.09	2	0.43	4	2	5
	*TBP*	0.946	8	2.327	8	1.45	8	2.5	8
	*β-ACT*	0.853	7	1.931	7	1.15	7	2.23	7
	*CypA*	0.767	6	1.539	6	0.86	6	2.02	6
Abiotic treatment	*GAPDH*	0.384	3	1.458	6	0.68	4	1.94	4
	*18S*	2.546	10	4.937	10	5.04	10	5.01	10
	*28S*	1.931	9	4.222	9	4.36	9	4.45	9
	*EF*	0.689	6	0.238	3	0.30	2	1.81	1
	*RPL12*	0.471	4	0.917	4	0.29	1	1.82	2
	*β-TUB*	0.938	7	0.160	1	0.81	5	2.00	5
	*ATPase*	1.084	8	0.160	2	0.93	7	2.08	7
	*TBP*	0.557	5	2.184	8	1.21	8	2.36	8
	*β-ACT*	0.350	1	1.760	7	0.88	6	2.07	6
	*CypA*	0.350	1	1.455	5	0.64	3	1.93	3

**Table 4:** Expression stabilities of the candidate reference genes of all samples.

Abiotic condition	Reference gene	geNorm		Normfinder		BestKeeper		△Ct	
		Stability	Rank	Stability	Rank	Stability	Rank	Stability	Rank
All samples	*GAPDH*	1.148	5	1.242	6	1.14	5	2.14	5
	*18S*	2.102	9	3.049	9	3.38	10	3.5	9
	*28S*	2.49	10	3.759	10	3.29	9	4.04	10
	*EF*	0.823	3	0.779	2	0.85	1	1.89	2
	*RPL12*	0.625	1	1.005	4	1.1	3	1.96	4
	*β-TUB*	0.97	4	0.209	1	1.37	7	1.88	1
	*ATPase*	1.408	7	0.926	3	1.28	6	2.14	6
	*TBP*	1.274	6	1.939	7	1.12	4	2.45	7
	*β-ACT*	1.68	8	2.437	8	2.35	8	2.94	8
	*CypA*	0.625	1	1.068	2	0.99	2	1.95	3

**Table 5:** The first three most stable reference genes recommended by RefFinder under various experimental conditions.

Biotic and abiotic condition		Reference gene		
Biotic factors	Developmental stages	*EF *	*CypA*	*β-TUB*
	Larval tissues	*EF*	*CypA*	*RPL12*
	Adult tissues	*EF*	*TBP*	*β-TUB*
	Densities	*RPL12 *	*β-TUB*	*EF*
	All biotic samples	*EF *	*β-TUB*	*CypA*
Abiotic stress	Photoperiod treatments	*EF *	*RPL12*	*GADPH*
	Temperature treatments	*β-TUB *	*EF*	*ATPase*
	All abiotic samples	*RPL12 *	*EF*	*CypA*
All samples		*EF *	*β-TUB*	*CypA*

## Data Availability

The data used to support the findings of this study are available from the corresponding author upon request.
